# Demographic and Health Status Differences Among People Aged 45 or Older With and Without Functional Difficulties Related to Increased Confusion or Memory Loss, 2011 Behavioral Risk Factor Surveillance System

**DOI:** 10.5888/pcd12.140429

**Published:** 2015-03-05

**Authors:** Lynda A. Anderson, Angela Deokar, Valerie J. Edwards, Erin D. Bouldin, Kurt J. Greenlund

**Affiliations:** Author Affiliations: Angela Deokar, Valerie J. Edwards, Kurt J. Greenlund, Division of Population Health, Centers for Disease Control and Prevention, Atlanta, Georgia; Erin D. Bouldin, VA Puget Sound Health Care System, Seattle, Washington. Dr Anderson is also affiliated with the Rollins School of Public Health, Emory University, Atlanta, Georgia.

## Abstract

We examined the demographic and health characteristics of people aged 45 years or older in 21 states with self-reported increased confusion or memory loss (ICML) (n = 10,583) by whether or not they also reported functional difficulties related to ICML. We used data from the 2011 Behavioral Risk Factor Surveillance System optional module on impact of cognitive impairment. After adjusting for demographic differences, we found that respondents with ICML and functional difficulties were significantly more likely than those with ICML and no functional difficulties to report frequent poor physical health, frequent poor mental health, limited activity due to poor physical or mental health, and a need for more help. Further understanding of the implications for long-term services and supports is needed.

## Objective

In response to national recognition of the importance of perceptions about cognitive functioning and its effect on functioning and well-being (1,2), several public health surveillance systems included questions on this topic. Questions about increased confusion or memory loss (ICML) were added to the Behavioral Risk Factor Surveillance System (BRFSS) in 2011. The objective of our study was to describe the demographic characteristics of people aged 45 years or older who reported functional difficulties related to ICML, compare these characteristics with the characteristics of those who did not report functional difficulties, and then compare the 2 groups on selected measures of health and well-being.

## Methods

BRFSS is a state-based random-digit–dialed telephone survey of noninstitutionalized US adults aged 18 or older that examines perceptions about health and health behaviors (3). In 2011, 21 states included the optional module on the impact of cognitive impairment. Our study focused on adults aged 45 years or older who responded yes to the question “During the past 12 months, have you experienced confusion or memory loss that is happening more often or is getting worse?” (Figure). Functional difficulties were assessed by asking respondents whether ICML either caused them to give up household activities or chores or interfered with the ability to work, volunteer, or engage in social activities (always, usually, or sometimes vs rarely or never). Additional questions addressed the need for support related to ICML and whether help was received.

**Figure:**
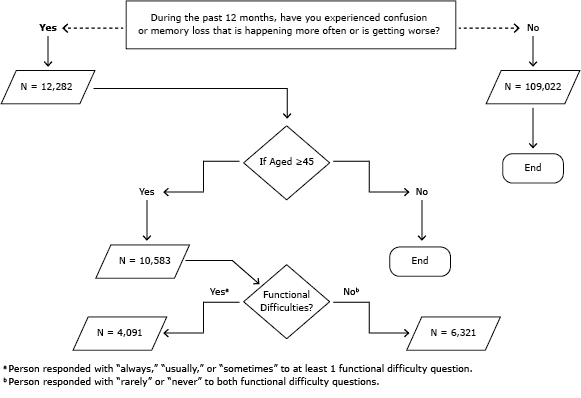
Optional module on the impact of cognitive impairment, Behavioral Risk Factor Surveillance System, in 21 states, January 1, 2011–December 31, 2011. Analysis conducted March–May 2013. The module is introduced with the following: “The next few questions ask about difficulties in thinking or remembering that can make a big difference in everyday activities. This does not refer to occasionally forgetting your keys or the name of someone you recently met, which is normal.”

The BRFSS core survey included questions on age, sex, race, ethnicity, education level, and employment status. Respondents were classified as living alone based on BRFSS screening procedures that determine household composition. Disability status was ascertained from reported activity limitations, use of special equipment, or both. Having 1 or more chronic condition was determined by asking about 6 conditions named in the BRFSS core survey: arthritis, asthma, cardiovascular disease, cancer, chronic obstructive pulmonary disease, and diabetes. Physically and mentally unhealthy days and activity-limited days were classified as being frequent if respondents reported 14 days or more in the previous month (4).

Data were analyzed using SAS 9.4 (SAS Institute, Inc) and Stata 12.1 (StataCorp LP) and accounted for the complex sampling design. Analyses were restricted to landline respondents because only 7 states included cellular telephone respondents in the module and evidence suggests that cellular telephone respondents differ from landline respondents (5). Weighted proportions and 95% confidence intervals (CIs) were generated for all variables. *P* values were based on survey design–corrected Pearson χ^2^ tests. Among respondents with ICML, we used complementary log–log regression to estimate the prevalence ratio (PR) of well-being for those with functional difficulties and those without such difficulties in 5 models, each adjusted for categorical age, race/ethnicity, and education (6).

## Results

The sample consisted of 10,583 (12.5% [weighted]; 95% CI, 11.9%–13.0%) respondents who reported ICML. Among these, 10,412 responded to the questions on functional difficulties; 42.9% were classified as ICML with functional difficulties and 57.1% as ICML with no functional difficulties. Respondents who reported ICML with functional difficulties tended to be younger and have less education than those who reported ICML and no functional difficulties (Table). We found no differences in the proportions of men and women between the 2 groups. Respondents with ICML and functional difficulties were more likely than those with ICML and no functional difficulties to be black or Hispanic, single (divorced/widowed/separated or never married), or unable to work; they were also more likely to have a disability, live alone, or have at least 1 chronic health condition.

**Table:** Demographic and Health Status Measures Among People With and People Without Functional Difficulties Related to Self-Reported Increased Confusion or Memory Loss, Persons Aged 45 or Older, 21 US States, BRFSS, 2011^a^

Characteristic	Increased Confusion or Memory Loss With Functional Difficulties^b^ (n = 4,091)	Increased Confusion or Memory Loss Without Functional Difficulties^b^ (n = 6,321)	All Respondents With Increased Confusion or Memory Loss (n = 10,412)
**Age^c^, y**			
45–54	43.0 (39.1–47.0)	30.9 (28.2–33.9)	36.1 (33.8–38.6)
55–64	31.5 (28.3–34.9)	26.9 (24.5–29.5)	28.9 (26.9–30.9)
65–74	11.7 (10.0–13.7)	21.7 (19.7–23.8)	17.4 (16.0–18.9)
75–84	10.1 (8.4–12.2)	15.8 (14.3–17.4)	13.4 (12.2–14.6)
≥85	3.7 (2.9–4.7)	4.6 (3.9–5.5)	4.2 (3.6–4.9)
**Sex**			
Male	44.5 (40.7–48.3)	47.4 (44.6–50.2)	46.1 (43.9–48.4)
Female	55.5 (51.7–59.3)	52.6 (49.8–55.4)	53.9 (51.6–56.1)
**Race/ethnicity** c			
Non-Hispanic white	59.3 (55.2–63.3)	74.4 (71.5–77.2)	68.0 (65.5–70.4)
Non-Hispanic black	14.3 (11.9–17.0)	6.7 (5.3–8.3)	9.9 (8.6–11.4)
Other non-Hispanic race or non-Hispanic multirace	8.9 (5.9–13.2)	6.8 (5.3–8.8)	7.7 (6.1–9.7)
Any race, Hispanic	17.6 (14.4–21.2)	12.1 (9.8–14.8)	14.4 (12.5–16.5)
**Education** c			
Less than high school	33.1 (29.2–37.3)	17.8 (15.4–20.5)	24.4 (22.1–26.8)
High school graduate	28.5 (25.4–31.8)	29.1 (26.7–31.6)	28.9 (26.9–30.9)
Some college	25.8 (22.8–29.1)	31.9 (29.4–34.4)	29.3 (27.3–31.3)
College graduate	12.6 (10.8–14.6)	21.1 (19.2–23.2)	17.5 (16.1–18.9)
**Marital status** c			
Married/partnered	39.5 (36.0–43.1)	57.5 (54.8–60.2)	49.7 (47.5–52.0)
Divorced/widowed/ separated	45.9 (42.2–49.7)	35.1 (32.6–37.7)	39.7 (37.6–41.9)
Never married	14.6 (11.0–19.1)	7.4 (6.0–9.2)	10.5 (8.7–12.7)
**Employment status** c			
Employed for wages or self-employed	18.3 (14.8–22.3)	35.1 (32.4–37.9)	27.9 (25.7–30.2)
Not employed	36.1 (32.8–39.7)	51.7 (48.9–54.5)	45.0 (42.8–47.2)
Unable to work	45.6 (41.9–49.4)	13.2 (11.3–15.4)	27.1 (25.1–29.2)
**Disability status** d			
Disability	81.3 (77.2–84.7)	53.1 (50.4–55.9)	65.2 (62.9–67.4)
No disability	18.7 (15.2–22.8)	46.9 (44.1–49.6)	34.8 (32.6–37.1)
**Lives alone** d			
Yes	28.7 (26.0–31.6)	24.0 (22.3–25.9)	26.0 (24.5–27.7)
No	71.3 (68.4–74.0)	75.9 (74.1–77.7)	73.9 (72.3–75.5)
**Has ≥1 chronic condition** c ^, ^ e			
Yes	84.3 (81.2–86.9)	75.9 (73.4–78.3)	79.5 (77.6–81.3)
No	15.7 (13.1–18.8)	24.1 (21.7–26.6)	20.5 (18.7–22.4)
**Had ≥14 days poor mental health in previous 30 days**			
Yes	53.5 (49.7–57.4)	20.2 (17.9–22.7)	34.3 (32.1–36.7)
No	46.5 (42.6–50.3)	79.8 (77.3–82.1)	65.6 (63.3–67.9)
**Had ≥14 days poor physical health in previous 30 days**			
Yes	54.4 (50.4–58.3)	28.4 (25.9–31.0)	39.5 (37.3–41.7)
No	45.6 (41.7–49.6)	71.6 (69.0–74.1)	60.5 (58.3–62.7)
**Had ≥14 days of limited activity in past 30 days**			
Yes	59.8 (55.7–63.7)	44.9 (42.2–47.7)	51.2 (48.9–53.5)
No	40.2 (36.3–44.3)	55.1 (52.3–57.8)	48.8 (46.5–51.1)
**Needs assistance related to increased confusion or memory loss**			
Yes	80.7 (76.8–84.1)	39.3 (36.5–42.1)	56.9 (54.6–59.1)
No	19.3 (15.9–23.1)	60.7 (57.9–63.5)	43.1 (40.8–45.4)
**Always or usually receives care or assistance related to increased confusion or memory loss**			
Yes	16.8 (14.3–19.7)	1.4 (0.9–2.1)	8.0 (6.8–9.3)
No	83.2 (80.3–85.7)	98.6 (97.8–99.1)	92.0 (90.7–93.2)

Abbreviation: BRFSS, Behavioral Risk Factor Surveillance System.

a All values are weighted percentages and 95% confidence intervals. All estimates are based on unweighted counts of at least 50 respondents. Columns may not sum to 100% because of rounding.

b Respondents were counted as having functional difficulties when they answered “always,” “usually,” or “sometimes” to 1 of 2 questions about whether increased confusion or memory loss interfered with their “ability to work, volunteer, or engage in social activities” or caused them to “give up household activities or chores” that they “used to do.”

c *P* < .001 for survey design–corrected Pearson χ^2^ test comparing people with and people without functional difficulties related to increased confusion or memory loss.

d *P* = .006 for survey design–corrected Pearson χ^2^ test comparing people with and people without functional difficulties related to increased confusion or memory loss.

e Chronic conditions are arthritis, asthma, cardiovascular disease (heart attack, angina or coronary heart disease, or stroke), cancer (excluding skin), chronic obstructive pulmonary disease, and diabetes.

Adjusted analyses showed that respondents with ICML and functional difficulties were significantly more likely than those with ICML and no functional difficulties to report frequent poor physical health (PR = 2.24, 95% CI, 1.92–2.61), frequent poor mental health (PR = 3.06; 95% CI, 2.56–3.66), and limited activity due to poor physical or mental health (PR = 3.07; 95% CI, 2.54–3.69). Respondents with ICML and functional difficulties also were significantly more likely than respondents with ICML and no functional difficulties to report needing help related to ICML (PR = 3.09; 95% CI, 2.71–3.53) and to always or usually receive care from a family member or friend related to cognitive decline (PR = 13.03; 95% CI, 7.91–21.44).

## Discussion

Although ICML is commonly regarded as a concern only among older adults, we found that those with ICML and functional difficulties tended to be younger than those with ICML and no functional difficulties. Studies of adults aged 60 or older found similar trends (7). Ensuring the quality and accessibility of health services is a core public health function. Eligibility for services is often age-dependent (8); our findings underscore a need to ensure assistance for people who have ICML and functional difficulties but who do not meet the present age-related eligibility requirements.

A growing body of evidence suggests a greater burden of multiple chronic health conditions among adults aged 60 years or older who report memory limitations than among those who report no such limitations (9,10). We found a greater prevalence of disability and reports of poor mental and physical health among respondents aged 45 or older with ICML and functional difficulties than among similarly aged respondents with ICML and no functional difficulties. Among people with ICML in general, and particularly among people with ICML and functional difficulties, a greater percentage reported an unmet need for assistance. The health and social consequences for informal care partners is an area for future study.

This study has several limitations. Data are from 21 states, and therefore the sample does not represent the United States as a whole. Although questions underwent multiple rounds of cognitive testing, ICML is self-reported, might be subject to recall bias, and has not been validated by clinical assessment. The study design is cross-sectional, and causality cannot be inferred. We examined functional limitations related to ICML but did not account for all causes of functional disabilities. BRFSS respondents were drawn from households with a landline telephone, thereby limiting the sample to those who can afford one, and the survey also excludes adults in noninstitutionalized settings.

Our findings highlight the association between physical and cognitive health among a large group of community-dwelling respondents. That people with ICML and functional difficulties were younger than those with ICML and no functional difficulties calls for examination of cognitive health issues at younger ages. Public health surveillance monitors trends, identifies disparities, and informs public health service needs and research gaps. Another key aspect of work in this area concerns the prevalence of ICML in households; Deokar and colleagues examine ICML in households using 2011 Behavioral Risk Factor Surveillance System data from 13 states (11). The inclusion of cognition into public health surveillance needs to be accompanied by further insight into public health implications. Greater understanding is needed of long-term support services at the intersection of cognition, mental health, and physical health.
